# Environmental exposure to perchlorate, nitrate, and thiocyanate in relation to biological aging in U.S. adults, a cross-sectional NHANES study

**DOI:** 10.3389/fpubh.2025.1518254

**Published:** 2025-03-18

**Authors:** Weiliang Kong, Yina Jin

**Affiliations:** Department of Respiratory and Critical Care Medicine, Key Laboratory of Respiratory Disease of Ningbo, The First Affiliated Hospital of Ningbo University, Ningbo, China

**Keywords:** perchlorate, nitrate, thiocyanate, biological aging, NHANES

## Abstract

**Background:**

Few studies have investigated the associations between perchlorate, nitrate, and thiocyanate (PNT) and biological aging. This study aimed to assess the association between PNT and biological aging among U.S. adults.

**Methods:**

Utilizing multivariable linear regression and restricted cubic splines (RCS), we analyzed urinary PNT levels’ impact on phenotypic age and biological age. Subgroup and sensitivity analyses were also conducted. Weighted Quantile Sum (WQS) and Bayesian Kernel Machine Regression (BKMR) models examined PNT mixtures.

**Results:**

8,368 participants were analyzed. Mean phenotypic age was 43.05 ± 0.48 years, mean biological age was 47.08 ± 0.4 years. Multivariable linear regression showed significant negative associations between higher PNT levels and phenotypic age (perchlorate *β* = −0.6, 95% CI: −0.93 to −0.27; nitrate *β* = −0.81, 95% CI: −1.19 to −0.42; thiocyanate *β* = −0.56, 95% CI: −0.77 to −0.34) after covariates adjusted. RCS demonstrated negative nonlinear relationships between PNT exposure and phenotypic age (nonlinear *p* values: 0.002, <0.001, and <0.001), with stable results in sensitivity analyses. Nitrate exposure showed a significant negative association with biological age (*β* = −0.78, 95% CI: −1.13 to −0.44), indicating a consistent negative linear relationship observed through RCS and remaining stable across sensitivity analyses. WQS regression revealed a negative association between the mixture and phenotypic age in both positive and negative directions, with a significant negative association with biological age in the negative direction. BKMR analysis revealed a negative association between PNT mixtures and phenotypic age, with nitrate and thiocyanate identified as the primary predictors of phenotypic age. No association found between PNT mixture and biological age.

**Conclusion:**

Individual or combined PNT are negatively associated with phenotypic age. High nitrate is associated with reduced biological age, showcasing consistent outcomes.

## 1 Introduction

Aging is a complex pathological and physiological process driven by a series of intricate biological mechanisms associated with deteriorating physiological systems, involving multiple dimensions of cells, tissues, and organs, and influenced by a range of environmental, biopsychosocial, and demographic factors (1). Aging also leads to adverse health outcomes, correlated with the occurrence and progression of a range of chronic diseases such as cardiovascular diseases (CVD), cancer, osteoporosis, neurodegenerative diseases, etc., and conversely, these adverse health outcomes may also impact aging (2). Existing methods for measuring biological aging vary significantly in their approaches and measurement focuses due to the complexity of the aging process (3). Currently developed measurement methods reflecting biological aging include, phenotypic age (4), biological age (5), leukocyte telomere length (6), and metabolic age score (7), etc. Nowadays, population aging has become a prominent global trend. According to the State of World Population Report 2023 (8), the current population aged 60 and above comprises 12.3%, a figure projected to increase to 22% by 2050. Aging brings about an unbearable burden of chronic diseases to humanity, resulting in enormous social and economic costs. Abundant epidemiological research has shown that biological aging is significantly influenced by environmental factors, such as Per- and polyfluoroalkyl substances (PFAS) (9), urinary levels of Cd and Mo (10), 3,5,6-trichloro-2-pyridinol (11), a mixture of benzene, toluene, ethylbenzene, and xylenes (12), and 2,5-Dichlorophenol (13). However, it remains unclear whether biological aging is influenced by perchlorates, nitrates, and thiocyanates (PNT). These compounds are known inhibitors of the sodium/iodide symporter (NIS), exerting endocrine-disrupting effects by influencing thyroid function (14).

Perchlorate occurs naturally and can be manufactured as rocket fuel (as an oxidizer) and utilized in airbags, fireworks, or fertilizers. Most perchlorates are soluble in water, exhibit high chemical stability. Individuals are primarily exposed to perchlorates through water and food (15). Thiocyanate, composed of sulfur, carbon, and nitrogen, is a group of compounds. It serves as a major metabolite in cigarette smoke, and is also present in cruciferous vegetables such as radishes, kale, and other leafy greens, as well as dairy products. Individuals are primarily exposed to thiocyanate through food and smoke (14). Nitrate refers to compounds derived from nitric acid. Its natural sources include biological nitrogen fixation and the reaction of nitric acid with minerals in rainwater (16). It is extensively used as an agricultural fertilizer, food preservative, and coloring agent (17). Over 80% of nitrate exposure in human comes from diet (water and vegetables) (17, 18). The urinary concentration is a commonly used indicator for assessing PNT exposure (19, 20). Currently, a few studies have reported potential health benefits from PNT exposure, including effects on blood pressure (21), obesity (22), renal function (23), cardiovascular disease (24), and mortality (25). While limited research suggests that higher perchlorate levels may adversely impact the serum anti-aging protein *α*-Klotho (26). Given these varying observations, further investigation into the relationship between PNT exposure and biological aging is warranted. Phenotypic age and biological age calculated from clinical observational data are considered relatively reliable predictors of biological aging outcomes. Thus, this study aims to explore the relationship between PNT exposure, and the selected biological aging using data from the National Health and Nutrition Examination Survey (NHANES).

## 2 Materials and methods

### 2.1 Design

**Figure 1:**
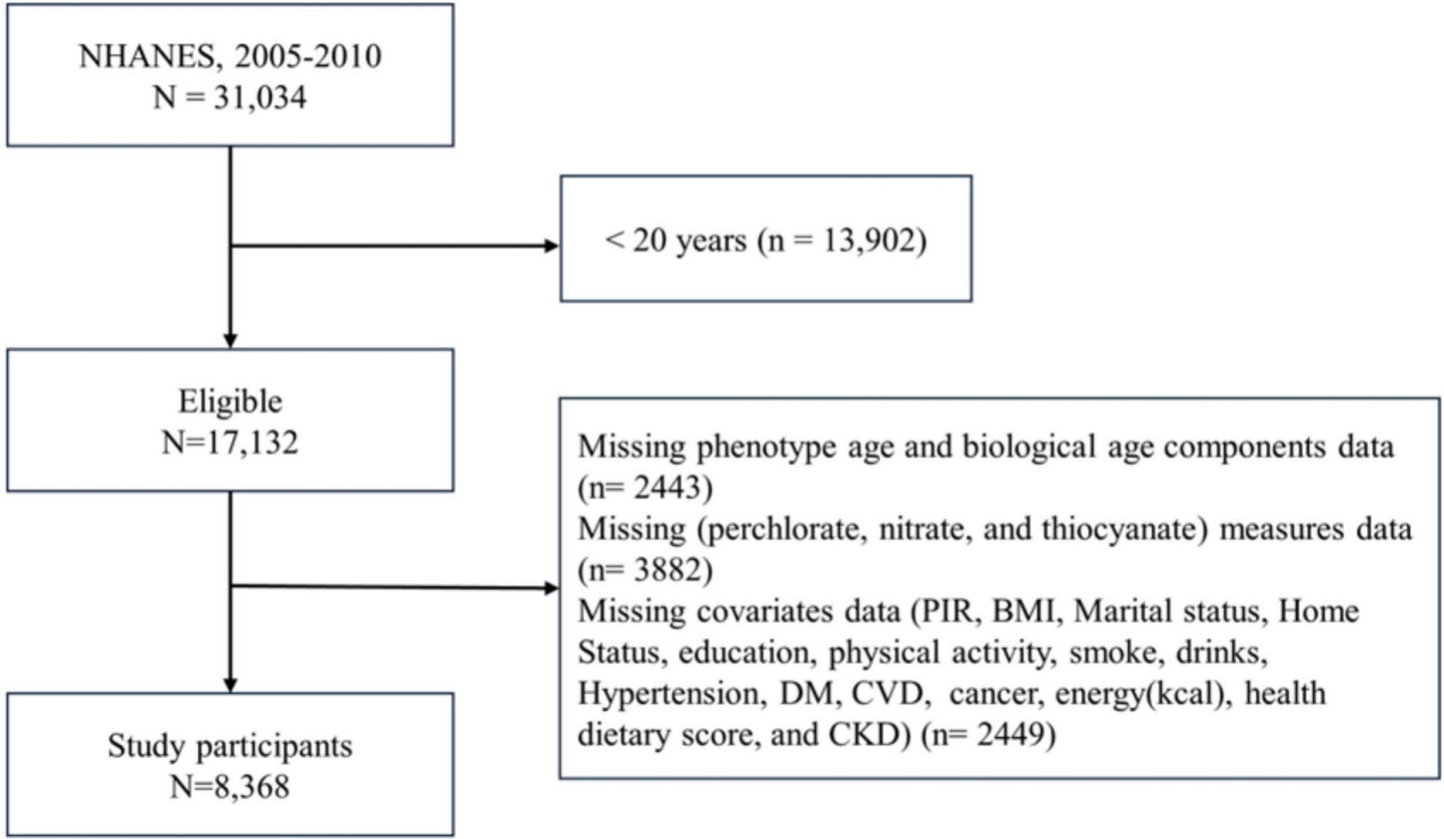
Participants flow chart.

The study utilized NHANES data spanning from 2005 to 2010, covering three consecutive survey cycles: NHANES 2005–2006, 2007–2008, and 2009–2010. Complete data of PNT have been available since 2005. However, C-reactive protein (CRP), a critical clinical indicator used to calculate phenotypic age, underwent a change in testing methodology starting in the 2011–2012 cycle, with the introduction of high-sensitivity CRP (hs-CRP) reporting. To maintain data consistency, data from the 2011–2012 cycle onwards have not been included. NHANES, operated by the CDC, is a comprehensive epidemiological survey program employing a complex, multi-stage sampling strategy to gather representative data on the health and nutrition of the U.S. population. Participants undergo questionnaire interviews, physical examinations, and biospecimen collection at mobile examination centers. For this study, participants aged 20 and above provided information on demographics, socio-economic factors, dietary intake, chronic diseases, and PNT levels during the 2005–2010 survey cycles. Following additional inclusion and exclusion criteria, 8,368 participants were included in the analysis. Further details are provided in Figure 1.

### 2.2 PNT exposure

At the Mobile Examination Center (MEC), biospecimens, including blood and urine samples, are collected from participants randomly assigned to morning, afternoon, or evening examinations. Fully voided urine specimens are collected from those aged 6 years and above using specimen cups. These biospecimens are processed, aliquoted, and stored at the MEC, refrigerated or frozen, before being transported to laboratories across the U.S. Ion chromatography-electrospray tandem mass spectrometry is utilized to measure PNT concentrations in urine, with urinary creatinine levels determined using automated colorimetric methods and Beckman Synchron AS/ASTRA clinical analyzers.

To ensure quality assurance, all collection materials and storage containers undergo preliminary screening, with three concentration gradient quality control materials prepared and inserted into each sample batch. Quality control samples are treated identically to test samples. For detailed laboratory information and quality control/quality assurance data, please refer to the NHANES website.^1^ Values below the limit of detection (LOD) are coded as the LOD value divided by the square root of 2. Subsequently, concentrations of chemicals in urine, after creatinine correction, are log-transformed using the natural logarithm to facilitate statistical analysis.

### 2.3 Biological aging

Biological aging is measured using two metrics: phenotypic age and biological age, which utilize different biomarkers and calculation methods.

The calculation of biological age is based on eight biomarkers (Ln-CRP, serum creatinine, glycated hemoglobin, serum albumin, serum total cholesterol, serum urea nitrogen, serum alkaline phosphatase, and systolic blood pressure) as outlined by Klemera (5, 27). The biomarkers and samples are denoted by the values *j* and *i*, respectively. The slope, intercept, and root mean square error of the regression of biomarkers on chronological age are represented by *k*, *q*, and *s* values. The variance explained by the regression of biomarkers on chronological age is depicted as $r_{j}^{2}$. *BA* represents biological age, *BA_E_* represents estimated biological age, and CA represents chronological age. *m* represents number of the biomarkers, and x represents the value of the biomarkers. The characteristic value $r_{\text{char}}$ for a set of *m* various correlation coefficients $r_{j}$ is determined as their weighted average, expressed by the following formula.

Klemera and Doubal Method (KDM) Biological Age formula:


$$BA_{E}=\frac{\sum_{j=1}^{m}\;\left(x_{j}-q_{j}\right)\left(\frac{k_{j}}{s_{j}^{2}}\right)}{\sum_{j=1}^{m}\;{\left(\frac{k_{j}}{s_{j}}\right)}^{2}}$$

$$r_{\text{char}}=\frac{\sum_{j=1}^{m}\;\frac{k_{j}}{s_{j}}r_{j}}{\sum_{j=1}^{m}\;\frac{k_{j}}{s_{j}}}$$

$$\begin{array}{l} s_{\text{BA}}^{2}={\frac{\sum_{j=1}^{n}\;\left(\left({\text{BA}}_{Ei}-{CA}_{i}\right)-\frac{\sum_{i=1}^{n}\;\left({\text{BA}}_{Ei}-{CA}_{i}\right)}{n}\right)}{n}}^{2}\\ -\left(\frac{1-r_{\text{char}}^{2}}{r_{\text{char}}^{2}}\right)\times \left(\frac{{\left({CA}_{\text{max}}-CA_{\text{min}}\right)}^{2}}{12m}\right) \end{array}$$

$$\text{Biological}\;\text{age}=\frac{\sum_{j=1}^{m}\;\left(x_{j}-q_{j}\right)\left(\frac{k_{j}}{s_{j}^{2}}\right)+\frac{CA}{s_{\text{BA}}^{2}}}{\sum_{j=1}^{m}\;{\left(\frac{k_{j}}{s_{j}}\right)}^{2}+\frac{1}{s_{\text{BA}}^{2}}}$$

Phenotypic Age was calculated using the following formula (4).


$$\text{Phenotypic}\;\text{Age}=141.50+\frac{\ln\left[-0.00553\times \ln\left(1-\text{MortalityScore}\right)\right]}{0.09165}$$

Where:


$$\text{MortalityScore}=1-\exp\left(\frac{-1.51714\times \exp\left(xb\right)}{0.0076927}\right)$$

And:


$$\begin{array}{l} xb=-19.907-0.0336\times \text{Albumin}\\ +0.0095\times \text{Creatinine}\\ +0.1953\times \text{Glucose}\\ +0.0954\times \text{LnCRP}\\ -0.0120\times \text{Lymphocyte}\;\text{Percentage}\\ +0.0268\times \text{Mean}\;\text{Cell}\;\text{Volume}\\ +0.3306\times \text{Erythrocyte}\;\text{Distribution}\;\text{Width}\\ +0.0019\times \text{Alkaline}\;\text{Phosphatase}\\ +0.0554\times \text{Leukocyte}\;\text{Count}\\ +0.0804\times \text{chronological}\;\text{age} \end{array}$$

(xb represents the linear combination of biomarkers from the fitted model).

### 2.4 Covariation

Our analysis encompassed a range of covariates previously demonstrated or assumed to be associated with PNT exposure and biological aging (26, 28). These covariates included age, as continuous variables or categorized as <40, 40–59, and ≥ 60 years; gender (female, male); racial/ethnicity background, reclassified as non-Hispanic black, non-Hispanic white, Mexican American, and others; poverty-to-income ratio (PIR), categorized as low (<1.3), middle (1.3–3.5), and high (>3.5); BMI according to World Health Organization (WHO) classifications: normal, overweight, and obese; marital status (reclassified as Widowed/Divorced/Separated, Married/Living with partner, and never married); education level (reclassified as college or higher, middle school or lower, and high school); home status (reclassified as owned or being purchased and rented); drinks (categorized as nondrinkers, moderate drinkers: 1–3 drinks/day, and heavy drinkers: ≥4 drinks/day); smoke (current, former, never); physical activity level [active, inactive, moderate, others (29)], presence of hypertension, diabetes mellitus (DM), CVD, and cancer. Hypertension and DM were determined through index measurements, medication usage, and self-reports, while CVD and cancer were self-reported. Additionally, we considered a healthy dietary score calculated using the Healthy Eating Index-2020 (HEI-2020) (30), as well as energy intake (kcal), derived from the mean of 2 days’ daily energy intake.

### 2.5 Statistical methods

Due to the complexity of NHANES sampling design, appropriate sample weights were used for analysis. For baseline characteristics, continuous variables were expressed as weighted means [standard errors (SE)], and categorical variables as weighted percentages (SE). Differences in continuous variable weighted means among female and male were assessed using ANOVA, while differences in categorical variable weighted percentages were assessed using Rao-Scott *χ*^2^ test.

Weighted linear regression analyses were conducted to examine the relationships between PNT exposure as continuous variables or categorized into quartiles and biological aging (including phenotypic age and biological age). Model 1 adjusted for age, sex, and race/ethnicity. Model 2 adjusted for age, sex, ethnicity, PIR, BMI, marital status, home status, education, physical activity, smoke, and drinks. Model 3 adjusted for age, sex, ethnicity, PIR, BMI, marital status, home status, education, physical activity, smoke, drinks, hypertension, DM, CVD, cancer, energy intake (kcal), healthy dietary score, and NHANES cycle. Additionally, restricted cubic spline (RCS) analysis was conducted to explore the dose–response relationship between PNT exposure (as continuous variables) and biological aging after adjusting for all confounding variables.

Furthermore, a series of sensitivity analyses were conducted to examine the robustness of the results. One analysis involved using covariate-adjusted creatinine rather than creatinine-corrected concentrations of analytes in statistical models. Since urinary creatinine correlates with some covariates such as age, sex, race/ethnicity, BMI, and eGFR, linear models were constructed to predict individual creatinine concentrations. Chemical concentrations were standardized by simulating the ratio of predicted to measured creatinine concentrations. Second, unweighted linear regression analyses were re-conducted to reassess the associations between each chemical exposure and the occurrence of biological aging.

In the second stage, Weighted Quantile Sum (WQS) regression was employed to investigate the effect of PNT mixture exposure on biological aging. Bootstrap method was utilized to assign individual weights to each pollutant, enabling the identification of the relatively important components within the mixture (31). The weights for each pollutant ranged from 0 to 1. This method not only better captures mixed exposures in real-life scenarios but also is more sensitive in identifying important predictors compared to univariate analysis. Specifically, in this study, the data were split into 40% training and 60% testing random samples, with bootstrap set at 10000.

Moreover, the Bayesian Kernel Machine Regression (BKMR) model was fitted to visualize the relationship curves between joint exposure to PNT mixture and the risk of biological aging. BKMR’s inherent advantage lies in its ability to flexibly fit exposure-response relationships, including potential nonlinear and non-additive effects, which are commonly encountered in environmental epidemiology (32). We modeled the exposure-response function with Gaussian distribution and ran 10,000 iterations using Markov Chain Monte Carlo (MCMC) algorithm. Key anions contributing most to the risk of biological aging were identified by calculating the conditional posterior inclusion probabilities (conPIPs). All statistical analyses were performed using R software (version 4.3.0).

## 3 Results

### 3.1 Characteristics of the study participants

Our study included 8,368 participants from NHANES 2005–2010 cycles. Table 1 summarizes the demographic and behavioral information by sex. The mean age of participants was 49.34 ± 0.42 years overall, with females at 47.94 ± 0.47 years and males at 46.70 ± 0.45 years (*p* = 0.002). The mean phenotypic age was 43.05 ± 0.48 years, with similar values for females and males (43.05 ± 0.55 versus 43.06 ± 0.52, *p* = 0.99). The mean biological age was 47.08 ± 0.4 years, with comparable values between females and males (46.83 ± 0.48 versus 47.35 ± 0.45, *p* = 0.15). Significant differences were found in PIR categories, BMI, marital status, education, smoke status, alcohol consumption (drinks), physical activity, energy intake, and healthy dietary score between females and males. Additionally, the distribution of PNT concentrations (creatinine-corrected) differed by sex. Females had higher perchlorate and nitrate concentrations compared to males (5.28 ± 0.14 versus 4.53 ± 0.11, *p* < 0.001, and 58608.83 ± 1567.37 versus 45555.46 ± 834.71, p < 0.001, respectively). Supplementary Tables 1-3 summarize the demographic and behavioral characteristics by quartiles of PNT concentrations. Association coefficient matrices are presented in Supplementary Figure 1, showing positive associations among all analytes (association coefficients 0.35 and 0.39).

**Table 1:** Characteristics of the study participants among U.S adults (NHANES2005-2010).

Variable	Total	Female	Male	*p* value
Age	47.34 (0.42)	47.94 (0.47)	46.70 (0.45)	<0.01
Phenotypic age	43.05 (0.48)	43.05 (0.55)	43.06 (0.52)	0.99
Biological age	47.08 (0.43)	46.83 (0.48)	47.35 (0.45)	0.15
Age groups				0.01
<40	35.20 (0.01)	33.62 (1.03)	36.88 (1.28)	
40–59	40.24 (0.02)	40.54 (0.98)	39.92 (1.16)	
≥60	24.56 (0.01)	25.84 (1.14)	23.20 (1.04)	
Race/ethnicity				0.09
Non-Hispanic White	73.50 (0.04)	73.22 (1.85)	73.79 (1.78)	
Non-Hispanic Black	9.85 (0.01)	10.52 (1.04)	9.14 (0.77)	
Mexican American	7.64 (0.01)	7.11 (0.84)	8.20 (0.94)	
Others	9.01 (0.01)	9.15 (0.96)	8.87 (0.96)	
PIR				0.01
Low	18.04 (0.01)	19.23 (0.94)	16.77 (1.12)	
Middle	37.05 (0.02)	37.63 (1.14)	36.44 (1.20)	
High	44.91 (0.03)	43.14 (1.65)	46.79 (1.45)	
BMI				<0.001
Normal	31.24 (0.01)	36.86 (1.06)	25.26 (1.08)	
Overweight	33.51 (0.01)	27.61 (0.78)	39.78 (1.10)	
Obesity	35.25 (0.02)	35.53 (0.99)	34.96 (1.30)	
Marital status				<0.001
Never married	15.17 (0.01)	14.01 (0.93)	16.41 (1.31)	
Married/Living with partner	66.58 (0.03)	62.70 (1.16)	70.70 (1.46)	
Widowed/Divorced/Separated	18.25 (0.01)	23.29 (0.71)	12.88 (0.77)	
Education				0.03
College or more	58.33 (0.02)	59.89 (1.27)	56.67 (1.51)	
Middle school or lower	5.41 (0.00)	5.11 (0.44)	5.73 (0.50)	
High school	36.26 (0.02)	35.00 (1.23)	37.60 (1.42)	
Home status				0.47
Owned or being bought	73.80 (0.04)	74.50 (1.31)	73.07 (1.45)	
Rented	24.47 (0.01)	23.91 (1.22)	25.07 (1.35)	
Smoke				<0.001
Now	21.37 (0.01)	18.96 (0.85)	23.92 (0.99)	
Former	26.02 (0.01)	21.87 (0.95)	30.44 (1.24)	
Never	52.61 (0.02)	59.17 (1.11)	45.64 (1.48)	
Drinks				<0.001
Nondrinkers	29.10 (0.01)	34.34 (1.41)	23.52 (1.03)	
1–3 drinks/day	54.90 (0.03)	57.25 (1.49)	52.41 (1.14)	
≥4 drinks/day	16.00 (0.01)	8.40 (0.50)	24.07 (1.10)	
Physical activity				<0.001
Active	39.60 (0.02)	32.36 (1.13)	47.29 (1.12)	
Inactive	26.24 (0.01)	28.37 (1.26)	23.97 (0.94)	
Moderate	13.67 (0.01)	14.91 (0.80)	12.34 (0.78)	
Others	20.50 (0.01)	24.37 (1.21)	16.39 (0.68)	
CVD	8.37 (0.01)	7.67 (0.65)	9.11 (0.60)	0.07
DM	12.65 (0.01)	12.21 (0.72)	13.11 (0.83)	0.33
Hypertension	36.21 (0.02)	35.03 (1.05)	37.46 (1.14)	0.05
Cancer	9.44 (0.01)	10.26 (0.65)	8.56 (0.76)	0.07
Energy (kcal)	2051.97 (15.67)	1735.62 (12.91)	2388.19 (24.54)	<0.001
Healthy Dietary Score	51.56 (0.31)	53.09 (0.40)	49.92 (0.39)	<0.001
Perchlorate^*^	4.92 (0.10)	5.28 (0.14)	4.53 (0.11)	<0.001
Nitrate^*^	52280.84 (973.87)	58608.83 (1567.37)	45555.46 (834.71)	<0.001
Thiocyanate^*^	2595.44 (87.45)	2617.96 (106.56)	2571.49 (111.62)	0.72
Year				0.79
2005–2006	34.01 (0.02)	33.88 (1.89)	34.15 (1.80)	
2007–2008	32.02 (0.02)	32.32 (1.92)	31.71 (1.75)	
2009–2010	33.96 (0.02)	33.80 (1.80)	34.14 (1.70)	

Weighted Mean +/− Se and ANOVA for continuous variables. Weighted %, mean (95% CI), and Rao-Scott *χ*^2^ test for categorical variables.

* Unit: μg/g creatinine.

### 3.2 Associations between PNT exposures and phenotypic age and biological age

**Figure 2:**
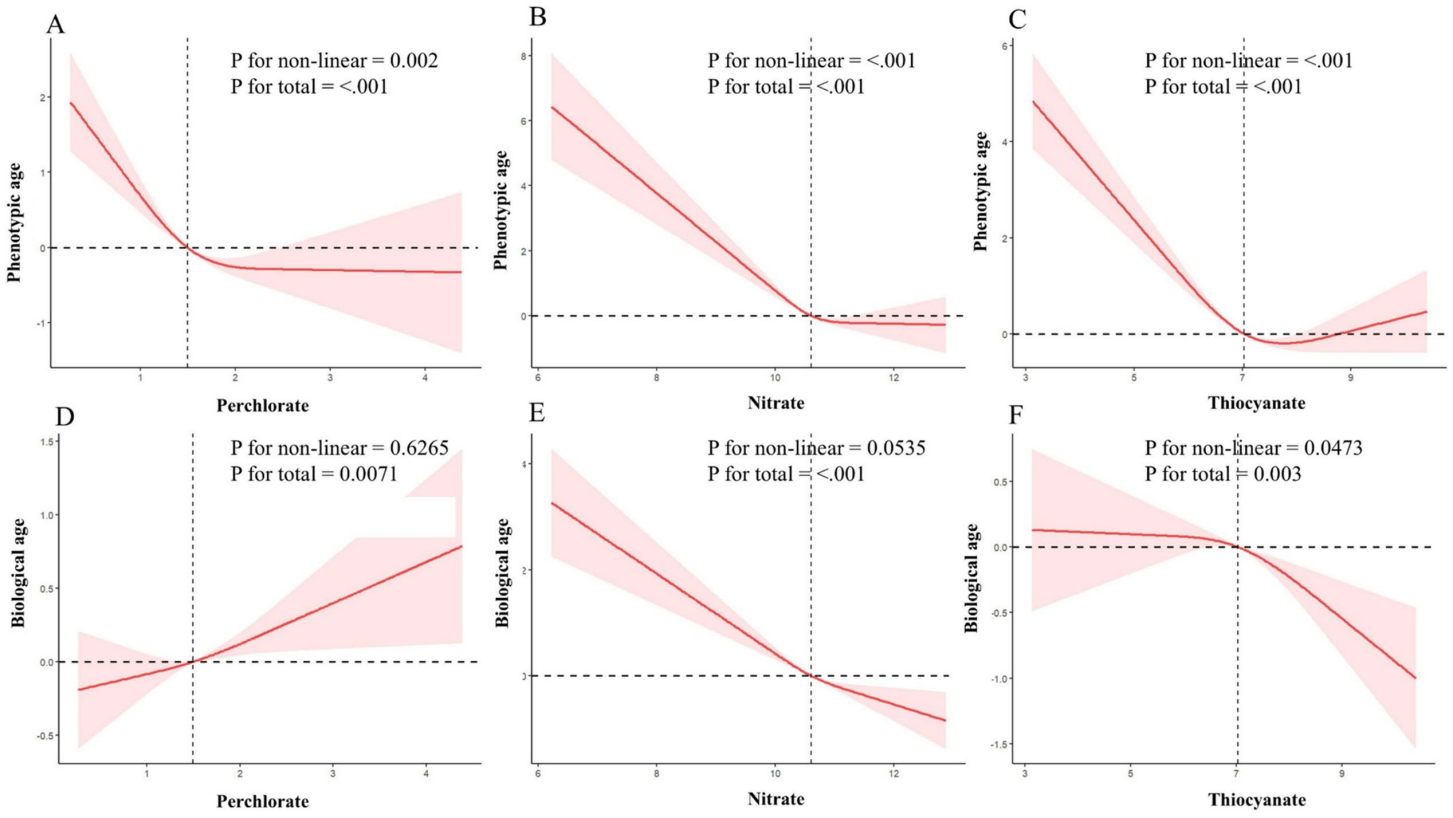
Dose–response relationship between perchlorate, nitrate, and thiocyanate, and their potential impact on phenotypic age and biological age in a sample of 8,368 US adults from NHANES 2005 to 2010. **(A–C)** Depict the associations between perchlorate, nitrate, and thiocyanate, respectively, with phenotypic age. **(D–F)** Illustrate the relationships with biological age. Red solid lines and red dotted line represent restricted cubic spline models and 95%CI, respectively. A multivariable linear regression model is used to estimate the fully adjusted coefficient in phenotypic age and biological age and corresponding 95% CI. Model was adjusted by age (continuous), sex, ethnicity, PIR, BMI, marital status, home status, education, physical activity, smoke, drinks, hypertension, DM, CVD, cancer, energy (kcal), health dietary score, and NHANES cycle.

The findings from the analysis of the association between each chemical exposure and the *β*-value of phenotypic age and biological age, while adjusting for relevant covariates, are summarized in Table 2. For phenotypic age, across almost all models (model 1, model 2, and model 3), a negative association was observed with high PNT concentration (ln-transformed). Specifically, when PNT exposures were treated as continuous variables, phenotypic age was significantly reduced with increasing PNT concentration after adjusting for specified covariates (model 3: perchlorate *β* = −0.6, 95% CI: −0.93 to −0.27; nitrate *β* = −0.81, 95% CI: −1.19 to −0.42; thiocyanate *β* = −0.56, 95% CI: −0.77 to −0.34). Additionally, the weighted *β* values with 95% CI for phenotypic age, categorized into quartiles of PNT exposures and accounting for relevant covariates, are also presented in Table 2. Following adjustments for all selected covariates using multiple linear regression (model 3), participants in the highest quartile of perchlorate exposure exhibited a significant decrease compared to the lowest quartile (*β* = −1.08, 95% CI: −1.48 to −0.68, P for trend <0.001). Similarly, for nitrate exposure (*β* = −1.00, 95% CI: −1.66 to −0.33, P for trend = 0.006) and thiocyanate (*β* = −1.52, 95% CI: −2.03 to −1.02, P for trend <0.001). Further exploration using RCS revealed a negative nonlinear relationship between PNT exposures and phenotypic age (P for non-linear = 0.002, <0.001, and < 0.001, respectively) as depicted in Figures 2A–C.

**Table 2:** Associations between perchlorate, nitrate, and thiocyanate exposures and phenotypic age and biological age.

Phenotypic age				Biological age		
	Model 1	Model 2	Model 3	Model 1	Model 2	Model 3
Perchlorate	−0.94 (−1.30, −0.59)	−0.6 (−0.93, −0.27)	−0.6 (−0.93, −0.27)	0.21 (0.04, 0.37)	0.25 (0.09, 0.41)	0.23 (0.05, 0.40)
Nitrate	−1.37 (−1.75, −0.99)	−0.64 (−0.95, −0.33)	−0.81 (−1.19, −0.42)	−0.9 (−1.10, −0.69)	−0.66 (−0.85, −0.47)	−0.56 (−0.76, −0.36)
Thiocyanate	0.06 (−0.12,0.25)	−0.62 (−0.85, −0.40)	−0.56 (−0.77, −0.34)	−0.26 (−0.36, −0.17)	−0.17 (−0.32, −0.03)	−0.16 (−0.31, −0.01)
Quartiles of perchlorate						
Q1	Ref.	Ref.	Ref.	Ref.	Ref.	Ref.
Q2	−1.00 (−1.45, −0.55)	−0.67 (−1.05, −0.29)	−0.71 (−1.13, −0.30)	−0.14 (−0.45,0.17)	−0.05 (−0.33, 0.23)	−0.06 (−0.35, 0.23)
Q3	−1.32 (−1.85, −0.80)	−0.92 (−1.35, −0.48)	−0.83 (−1.22, −0.44)	0.03 (−0.27,0.33)	0.12 (−0.16, 0.39)	0.15 (−0.14, 0.44)
Q4	−1.72 (−2.24, −1.20)	−1.16 (−1.57, −0.75)	−1.08 (−1.48, −0.68)	0.36 (0.10, 0.61)	0.45 (0.23, 0.68)	0.44 (0.20, 0.69)
P for trend	<0.001	<0.001	<0.001	0.001	<0.001	<0.001
Quartiles of nitrate						
Q1	Ref.	Ref.	Ref.	Ref.	Ref.	Ref.
Q2	−1.11 (−1.63, −0.60)	−1.11 (−1.57, −0.64)	−0.78 (−1.27, −0.29)	−0.66 (−0.90, −0.41)	−0.54 (−0.80, −0.28)	−0.39 (−0.66, −0.12)
Q3	−1.32 (−1.86, −0.78)	−1.41 (−1.96, −0.87)	−1.09 (−1.65, −0.52)	−0.94 (−1.33, −0.56)	−0.7 (−1.07, −0.33)	−0.55 (−0.90, −0.21)
Q4	−1.71 (−2.31, −1.11)	−1.37 (−1.99, −0.75)	−1.00 (−1.66, −0.33)	−1.33 (−1.68, −0.98)	−0.92 (−1.24, −0.59)	−0.78 (−1.13, −0.44)
P for trend	<0.001	<0.001	0.006	<0.001	<0.001	<0.001
Quartiles of thiocyanate						
Q1	Ref.	Ref.	Ref.	Ref.	Ref.	Ref.
Q2	−1.35 (−1.83, −0.86)	−1.31 (−1.80, −0.83)	−1.18 (−1.64, −0.71)	−0.34 (−0.62, −0.07)	−0.29 (−0.55, −0.03)	−0.24 (−0.49, 0.02)
Q3	−1.58 (−2.06, −1.10)	−1.71 (−2.25, −1.17)	−1.57 (−2.07, −1.07)	−0.21 (−0.50, 0.09)	−0.09 (−0.43, 0.24)	−0.01 (−0.30, 0.28)
Q4	−0.08 (−0.52, 0.36)	−1.77 (−2.28, −1.27)	−1.52 (−2.03, −1.02)	−0.72 (−1.02, −0.43)	−0.43 (−0.88, 0.03)	−0.40 (−0.86, 0.07)
P for trend	0.398	<0.001	<0.001	<0.001	0.125	0.159

Model 1: adjusted for age, sex and race/ethnicity.

Model 2: adjusted for age, sex, ethnicity, PIR, BMI, marital status, home status, education, physical activity, smoke, and drinks.

Model 3: adjusted for age, sex, ethnicity, PIR, BMI, marital status, home status, education, physical activity, smoke, drinks, hypertension, DM, CVD, cancer, energy (kcal), health dietary score, and NHANES cycle.

Ref.: reference.

For biological age, when PNT exposures were treated as continuous variables, positive associations were found with perchlorate exposure and biological age (*β* = 0.23, 95% CI: 0.05 to 0.40), while negative associations were observed with nitrate and thiocyanate exposures (*β* = −0.56, 95% CI: −0.76 to −0.36 and *β* = −0.16, 95% CI: −0.31 to −0.01). Upon further categorization of PNT exposures into quartiles, significant positive association was observed for the highest quartile of perchlorate exposure compared to the lowest quartile (*β* = 0.44, 95% CI: 0.20 to 0.69, P for trend <0.001), and a negative association for nitrate exposure (*β* = −0.78, 95% CI: −1.13 to −0.44, P for trend <0.001). No significant difference was found for thiocyanate quartiles in biological age (*β* = −0.40, 95% CI: −0.86 to 0.07, P for trend = 0.159). Figures 2D–F further illustrates the dose–response relationships between PNT exposures and biological age, showing a positive linear relationship for perchlorate (P for non-linear = 0.6265, Figure 2D), a negative linear relationship for nitrate (P for non-linear = 0.0535, Figure 2E), and a nonlinear relationship for thiocyanate exposures (P for non-linear = 0.0473, Figure 2F).

The results of stratified and interaction analyses of phenotypic age are presented in Supplementary Figure 2. In most sub-samples, higher levels of perchlorate and thiocyanate exposures corresponded with a decrease in phenotypic age, consistent with the primary results. However, differences were noted in some subgroups; notably, higher nitrate levels showed significant differences between age groups (<40 years vs. 40–59 and ≥ 60 years, P for interaction <0.001) and physical activity groups (P for interaction = 0.01). Similarly, increased thiocyanate levels showed significant differences in age groups, physical activity intensity groups, and smoking status groups (P for interaction <0.001, 0.01, and 0.02, respectively).

Supplementary Figure 3 presents the results of subgroup analysis for perchlorate and nitrate exposures and biological age. We found significant differences in high perchlorate exposure across gender groups (P for interaction = 0.002), while high nitrate exposure showed significant differences across age, race, PIR, physical activity, smoke, and drinks groups (P for interaction <0.001, 0.02, 0.01, 0.01, 0.04, and 0.049, respectively).

### 3.3 Associations between co-exposure to PNT mixture and phenotypic age and biological age

**Figure 3:**
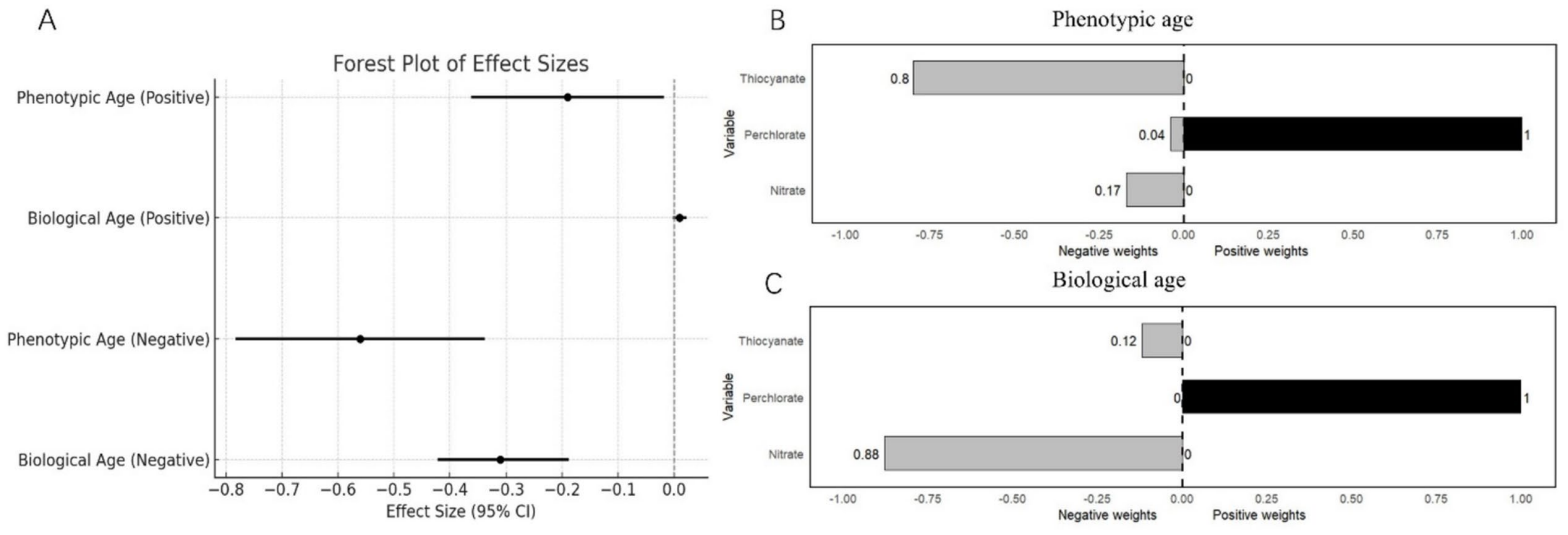
**(A)** Weighted quantile sums regression model for the associations of perchlorate, nitrate, and thiocyanate mixture with biological aging. **(B)** Weights from weighted quantile sum regression for the mixture and phenotypic age. Weights from weighted quantile sum regression for the mixture and biological age.

After adjusting for selected confounders, WQS regression revealed contrasting associations between the mixture and aging markers. For the positive direction of the mixture, there was a negative association with phenotypic age (*β* = −0.19, 95% CI: −0.36 to −0.02, *p* = 0.03), while no significant relationship was observed with biological age (*β* = 0.01, 95% CI: −0.00 to 0.02, *p* = 0.11). Conversely, for the negative direction of the mixture, phenotypic age showed a stronger negative association (*β* = − 0.56, 95% CI: −0.78 to −0.34, *p* < 0.0001), as did biological age (*β* = −0.31, 95% CI: −0.42 to −0.19, *p* < 0.0001). The estimated weighted results for each chemical exposure based on WQS regression are shown in Figure 3.

**Figure 4:**
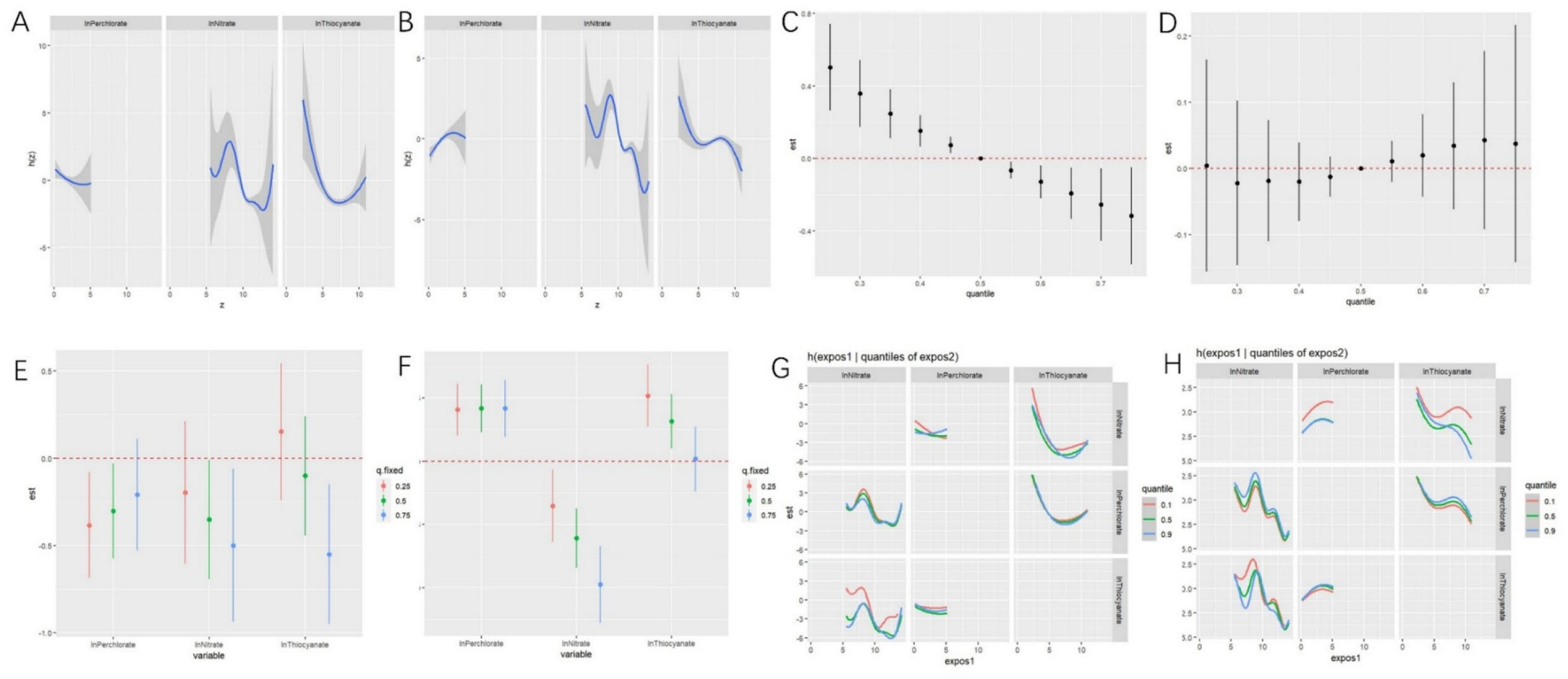
Associations between perchlorate, nitrate, and thiocyanate mixture and phenotypic age and biological age by Bayesian kernel machine regression (BKMR) for adults in NHANES 2005–2010. Univariate exposure–response functions between exposure to perchlorate, nitrate, and thiocyanate and phenotypic age **(A)** and biological age **(B)** calculated by the BKMR model. Joint effect of perchlorate, nitrate, and thiocyanate mixture on phenotypic age **(C)** and biological age **(D)** calculated by the BKMR model. Single chemical-exposure effect (95% CI) to phenotypic age **(E)** and biological age **(F)** when other chemicals were fixed at a specific quantile (25th, 50th, 75th). Bivariate exposure-response relationship between three anions and risk of phenotypic age **(G)** and biological age **(H)** (a visualization for evaluating interactions). These models were all adjusted for age (continuous), sex, ethnicity, PIR, BMI, marital status, home status, education, physical activity, smoke, drinks, hypertension, DM, CVD, cancer, energy (kcal), health dietary score, and NHANES cycle.

BKMR analyses indicated a negative univariate exposure-response relationship between perchlorate and phenotypic age, with a monotonic decreasing trend. Nitrate exhibited a wavelike curve variation, while thiocyanate showed a U-shaped relationship (Figure 4A). Figure 4B displayed a positive univariate exposure-response relationship between perchlorate and biological age, with an inverted U-shaped curve, whereas nitrate and thiocyanate were generally negatively associated with biological age, showing a wavelike downward trend.

Overall effects showed the distribution of high PNT mixtures on phenotypic age, as depicted in Figure 4C. Specifically, when the concentration of the chemical mixture was set at the 55th percentile, compared to the 50th percentile, it may have a significant overall impact on phenotypic age. However, we did not find significant effects on biological age (Figure 4D).

As depicted in Figure 4E, when we held other chemicals at different percentiles (25th, 50th, and 75th percentiles), we observed negative associations between PNT and Phenotypic age, except for thiocyanate when other chemicals were fixed at the 25th percentile. Figure 4F illustrated that when we held other chemicals at different percentiles (25th, 50th, and 75th percentiles), perchlorate and thiocyanate were positively associated with biological age, while nitrate was negatively associated with biological age.

Figure 4G illustrated the potential interaction between perchlorate and nitrate for Phenotypic age. BKMR analyses also revealed that nitrate and thiocyanate had the highest posterior inclusion probability (1.00), followed by perchlorate (0.4818). However, no potential interaction was found for biological age (Figure 4H). Then, BKMR analyses indicated that PNT had the same posterior inclusion probability (1.00).

### 3.4 Sensitivity analyses

We conducted two sensitivity analyses: one involved using covariate-adjusted creatinine instead of creatinine-corrected concentrations of analytes in linear regression models (Supplementary Table 4), and the other re-estimated using unweighted linear regression models (Supplementary Table 5). Both Supplementary Tables 4, 5 demonstrate that the relationships between PNT exposures and phenotypic age are consistent with the main results. Similarly, the relationship between nitrate exposure and biological age aligns with the main results. However, in both sensitivity analyses, although perchlorate exposure is positively associated with biological age, the differences in results are no longer significant. In the second sensitivity analysis, we observed a significant negative association between thiocyanate exposure and biological age. When thiocyanate was treated as a continuous variable, *β* = −0.29, 95% CI: −0.39 to −0.19, and when thiocyanate was categorized into quartiles, compared to the lowest quartile, the highest quartile of thiocyanate exposure still led to a decrease in biological age, with a β of −0.46, 95% CI: −0.78 to −0.14, P for trend <0.001.

## 4 Discussion

Based on our current knowledge, this study represents the inaugural investigation into the relationship between PNT exposure and biological aging. Our preliminary findings reveal a significant negative association between higher levels of individual and combined PNT exposure and phenotypic age, with individual PNT exposures showing a nonlinear relationship with phenotypic age. Nitrate and thiocyanate were identified as the primary drivers of the combined effect. Sensitivity analyses further underscored the robustness of these associations. However, the significant association between PNT exposure and biological age was noticed only with individual PNT, particularly nitrate, which exhibited a linear negative relationship and confirmed by sensitivity analyses. While WQS regression indicated a significant negative association between combined PNT exposure and biological age, BKMR analysis suggested no such relationship.

The aging process is influenced by both genetic and epigenetic factors (1). Currently, 12 existing features have been identified, such as genomic instability, telomere attrition, epigenetic alterations, loss of proteostasis, disabled macroautophagy, deregulated nutrient-sensing, mitochondrial dysfunction, cellular senescence, stem cell exhaustion, altered intercellular communication, chronic inflammation, and dysbiosis (1). These characteristics provide a multifaceted understanding of biological aging. We can assess biological aging through various methods, among which phenotypic age and biological age derived from serum biomarkers and clinical features are widely used in clinical practice [(33), pp. 1999–2018; (34)]. Levine et al. (4) identified actual age and nine clinical blood markers using a penalized Cox regression model. They then constructed phenotypic age using a proportional hazards model based on the Gompertz distribution. This approach showed a high association with actual age (0.94) and demonstrated strong predictive power for mortality, age-related diseases, comorbidity, and decline in physical function. The clinical biomarkers utilized in this method provide a comprehensive consideration of clinical presentation and functional status. In contrast, the KDM biological age demonstrates superior predictive ability for lifespan (5). This is because the relative weights of different markers in its calculation are determined by predicting actual age rather than assessing the risk of disease or mortality. Consequently, this method potentially emphasizes cellular aging and metabolic health. Therefore, there are notable distinctions between these two approaches.

In this study, we employed two of the most widely used multiple-pollutant models in epidemiology—WQS and BKMR—to investigate the health effects of multi-pollutant mixtures. Our results revealed significant associations between PNT (perchlorate, nitrate, and thiocyanate) and aging markers, underscoring the complexity of their combined effects. The WQS regression demonstrated contrasting effects depending on the exposure direction. Specifically, the positive direction of the mixture showed a mild negative association with phenotypic age, while no significant association was observed with biological age. Conversely, the negative direction of the mixture exhibited stronger negative associations with both phenotypic and biological age, suggesting that a negatively skewed exposure profile may exerts a more pronounced impact on aging markers. BKMR further emphasized the nuanced exposure-response relationships among individual components. Univariate analyses indicated a monotonic negative association between perchlorate and phenotypic age, while nitrate and thiocyanate displayed non-linear patterns, including wavelike and U-shaped trends. For biological age, perchlorate followed an inverted U-shaped curve, whereas nitrate and thiocyanate generally showed negative associations with wavelike patterns. These findings highlight the highly specific relationships between individual pollutants and aging markers, which are influenced by threshold effects and dose interactions. Overall effect analyses and interaction models reinforced the distinct roles of these chemicals. Higher percentile exposure to PNT mixtures may significantly impacted phenotypic age, while the effects on biological age were limited. This observation underscores the heightened sensitivity of phenotypic age as a biomarker of environmental exposures. Notably, thiocyanate exhibited distinct behavior under certain conditions, highlighting potential chemical-specific effects within mixture models.

Given the lack of prior consensus regarding the most appropriate model to evaluate multi-pollutant effects, our use of multiple statistical approaches helps mitigate the limitations of any single method and provides a more comprehensive perspective on the associations observed. While the underlying mechanisms of these mixture effects remain to be fully elucidated, findings from single-pollutant studies may offer partial explanations for the observed associations.

### 4.1 Nitrate and biological aging

High concentrations of nitrates have shown potential beneficial effects on both phenotypic and biological aging. Previous research highlights that nitrates positively influence various health outcomes. For example, Li et al. found that higher nitrate exposure is associated with improved kidney function (23), Xu et al. reported a negative association between PNT and hypertension (21), higher urinary nitrate levels have been associated with reduced obesity risk (22, 35), and elevated urinary nitrate levels are associated with a reduced prevalence of cardiovascular disease, congestive heart failure, and stroke (24). These health pathways are integral components of the aging process itself. Some studies have indirectly revealed the mechanisms of nitrate’s effects. The beneficial effects of nitrate on cardiovascular, renal, and metabolic function may be related to the nitrate-nitrite-nitric oxide (NO) pathway and the more classical L-arginine-NO synthase (NOS) pathway (53). NO can regulate vascular homeostasis, neurotransmission, and host defense (36). Moreover, Li and colleagues showed that treatments involving inorganic nitrate and nitrite mitigate renal fibrosis by targeting oxidative stress and lipid metabolism (37). These mechanisms, particularly through their influence on vascular homeostasis and oxidative stress—two key aspects of aging—underscore nitrate’s role in slowing the aging process (38, 39). Our research further shows that age and physical activity may modify the relationship between nitrates and phenotypic age. Additionally, factors such as race, income, physical activity, smoking, and alcohol consumption may influence the association with biological age. These variations could stem from differing physiological states and lifestyle factors, which may affect individuals’ sensitivity to nitrate exposure (40). Moreover, the source of nitrates—whether from vegetables or contaminated water—could result in different levels of exposure, leading to varying outcomes.

### 4.2 Thiocyanate and aging

Thiocyanate exhibits a nonlinear negative association with phenotypic age and serves as a primary driver of this combined effect. Evidence suggests that thiocyanate plays a protective role in the respiratory tract by exhibiting anti-inflammatory properties and reducing bacterial load (41). Animal and cell studies have demonstrated that thiocyanate can protect against harmful accumulations of hydrogen peroxide (H₂O₂) and hypochlorous acid (OCl) (42) or inhibit inflammation by reducing neutrophil infiltration and glutathione sulfonamide levels in cystic fibrosis, which is characterized by chronic infection and airway inflammation (43). These pathways, which influence respiratory health and inflammation—such as modulating inflammation and reducing bacterial burden—not only contribute to maintaining airway function but are also integral to the aging process, as dysregulated inflammation and cellular damage are hallmark features of aging (44). In addition, high thiocyanate levels may also associated with improved kidney function (23), so other potential mechanisms influencing the aging process cannot be ruled out. But notably, these studies primarily consider thiocyanate from dietary. Another significant source of thiocyanate is tobacco (45). For smokers, exposure to thiocyanate primarily comes from the metabolite cyanide found in cigarette use (46). High doses of thiocyanate can cause necrosis, and extremely high plasma levels are toxic (47, 48). This characteristic may explain why Thiocyanate exhibits a nonlinear negative association with phenotypic age and why smoking modifies the link between thiocyanate and aging. Observations also indicate that older adults and those engaging in moderate physical activity may benefit more from thiocyanate exposure, this may be due to changes in their metabolic and inflammatory responses. Age-related alterations in oxidative stress regulation (49), combined with immunomodulatory effects from physical activity (50), may modulate the effects of thiocyanate on aging.

### 4.3 Perchlorate and aging

Perchlorate shows a negative association with phenotypic age. Although a positive association is observed with biological age, the results are not consistent in sensitivity analyses. Limited literature indicates that perchlorate, as a endocrine disruptor, is associated with reduced serum parathyroid hormone levels (51). Reduced parathyroid hormone levels are independently associated with blood pressure and the presence of hypertension or pre-hypertension, suggesting that perchlorate may affect blood pressure through its impact on parathyroid hormone secretion, thereby indirectly influencing the aging process (52).

Our study has several strengths. Firstly, the analysis was conducted in a large, nationally representative population, and we constructed various statistical models, including traditional linear regression and RCS, as well as WQS, BKMR models, and various sensitivity analyses. As mentioned earlier, this is the first attempt to explore the relationship between PNT (individually and in combination) and two representative biological aging indicators among US adults. On the one hand, the application of these statistical strategies highlights the fact that people are frequently exposed to multiple pollutants in real life. On the other hand, integrating the advantages and disadvantages of various multi-pollutant approaches helps us better understand their combined effects and draw more reliable conclusions. However, there are still some limitations. Firstly, due to the cross-sectional nature of this study, causality cannot be inferred. Secondly, a decrease in glomerular filtration rate may reduce the urinary excretion of chemicals, potentially leading to reverse causality. Thirdly, the measurement of PNT is based on single-spot urine samples, although they still have considerable time reliability, the possibility of misclassification of exposure to these non-persistent chemical pollutants cannot be ruled out. Additionally, as NHANES employs complex sampling analysis, WQS and BKMR models are limited in addressing this sampling method, thereby restricting the scope of mixture analysis. Finally, due to the characteristics of the NHANES database, the confounding effects of some unmeasured factors were not considered.

## 5 Conclusion

In summary, our study reveals a negative association between PNT exposure, both individually and combined, and phenotypic age. The strong negative association between nitrate exposure and biological age is particularly notable. It’s important to note that our findings are based on PNT exposure levels in the general U.S. population, and we cannot clearly determine the sources of PNT exposure. This underscores the need for external validation and further exploration across different populations and various exposure sources. Furthermore, given the complexity of biological aging assessment and the importance of understanding aging comprehensively through diverse indicators and methodologies, there is a critical need for additional prospective cohort studies and carefully designed toxicological experiments to elucidate the causality of these relationships and uncover underlying mechanisms.

## Data Availability

Publicly available datasets were analyzed in this study. This data can be found here: data from NHANES collection was sponsored by the CDC, and are publicly available (https://wwwn.cdc.gov/nchs/nhanes/Default.aspx).

## Footnotes

^1^ https://www.cdc.gov/nchs/nhanes/index.htm
