# Supplementary material for: Environmental exposure to perchlorate, nitrate, and thiocyanate in relation to biological aging in U.S. adults, a cross-sectional NHANES study
Source: Front Public Health. 2025 Mar 18;13:1518254. doi: 10.3389/fpubh.2025.1518254 (PMC11958956; doi:10.3389/fpubh.2025.1518254)
Supplement: Supplementary file 1 [file Supplementary_file_1.docx]

**Online Supplementary Material**


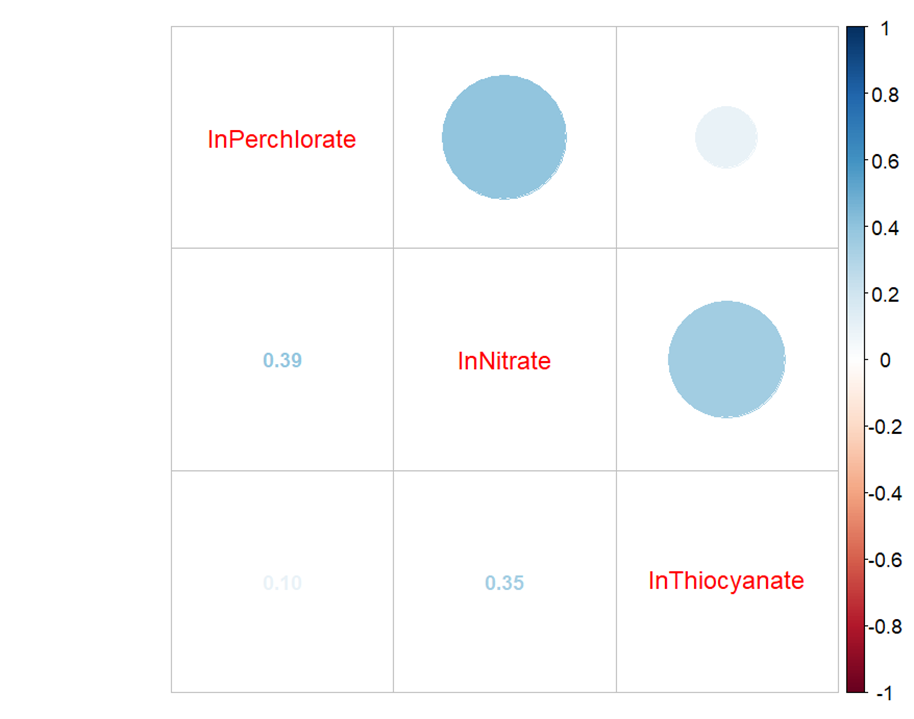


**Supplementary Figure 1.** Spearman Analysis of the three Substances (perchlorate, nitrate, and thiocyanate)


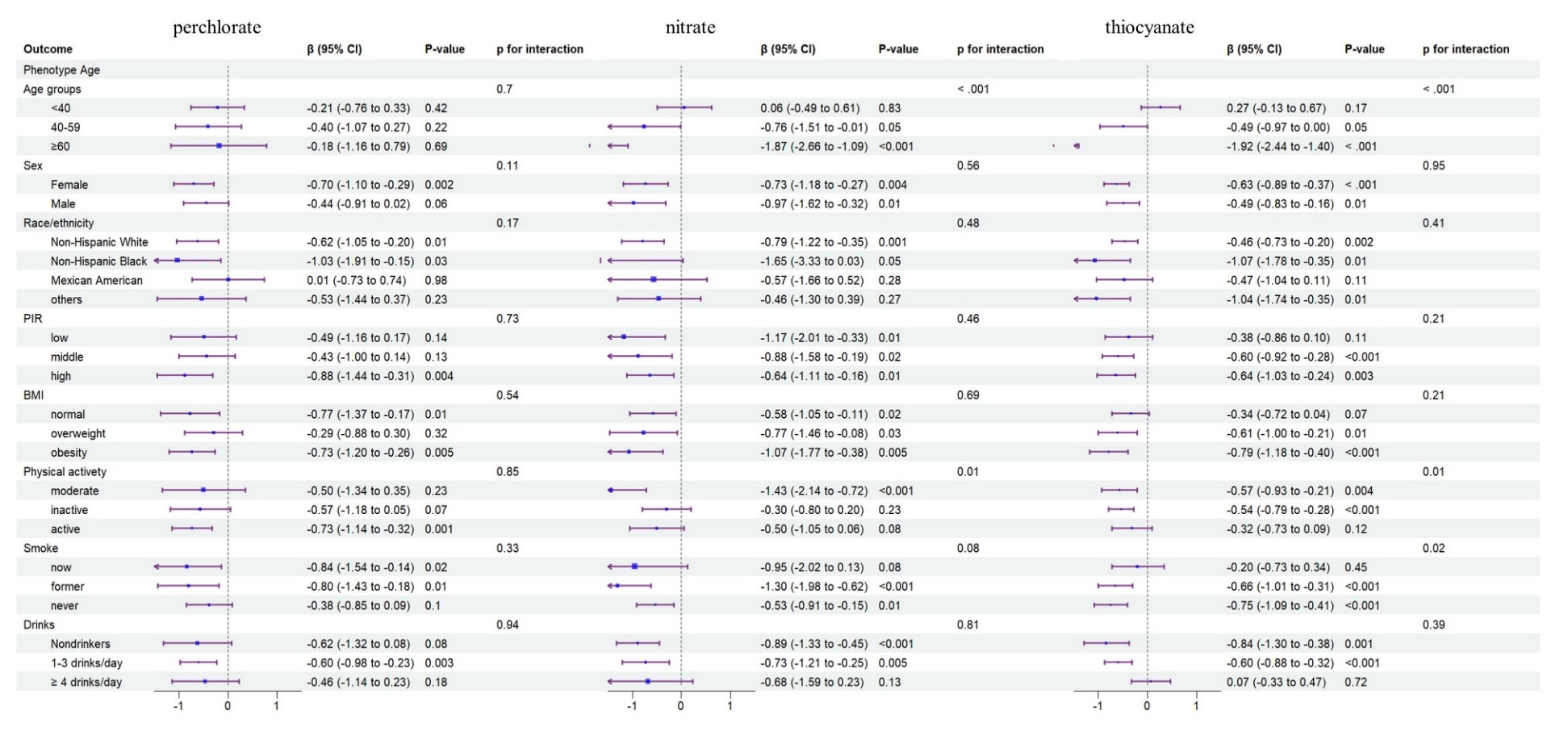


**Supplementary Figure 2**. Subgroup and interaction analyses of perchlorate, nitrate, and thiocyanate exposures and phenotypic age. Models were adjusted for age, sex, ethnicity, PIR, BMI, marital status, home status, education, physical activity, smoke, drinks, hypertension, DM, CVD, cancer, energy (kcal), health dietary score, and NHANES cycle. The subgroup variable was not included in same subgroup analysis.


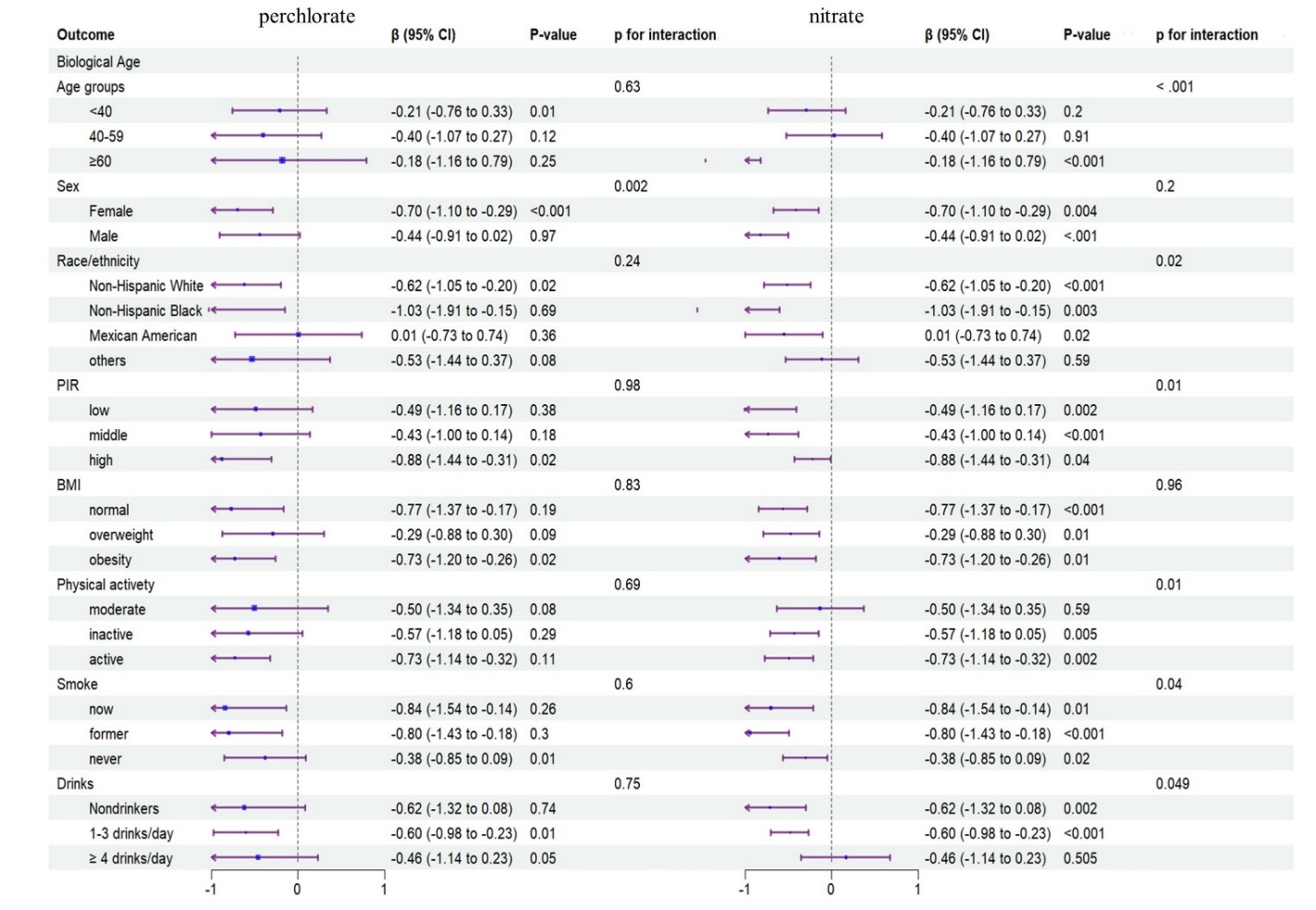


**Supplementary Figure 3**. Subgroup and interaction analyses of perchlorate and nitrate exposures and biological age. Models were adjusted for age, sex, ethnicity, PIR, BMI, marital status, home status, education, physical activity, smoke, drinks, hypertension, DM, CVD, cancer, energy (kcal), health dietary score, and NHANES cycle. The subgroup variable was not included in same subgroup analysis.

**Supplementary Table 1.** Characteristics of the study participants by quartiles of perchlorate.

|  | **Quartiles of** **Perchlorate** | | | |  |
| --- | --- | --- | --- | --- | --- |
| **Variable** | Q1 | Q2 | Q3 | Q4 | P value |
| **Phenotypic age** | 40.04(0.68) | 42.52(0.61) | 44.08(0.68) | 45.40(0.67) | **< .001** |
| **Biological age** | 43.33(0.60) | 46.41(0.61) | 48.26(0.53) | 50.12(0.57) | **< .001** |
| **Age** | 43.22(0.61) | 46.80(0.60) | 48.73(0.56) | 50.38(0.55) | **< .001** |
| **Age groups** |  |  |  |  | **< .001** |
| <40 | 45.55(1.80) | 36.31(1.55) | 31.86(1.42) | 27.70(1.46) |  |
| 40-59 | 37.82(1.45) | 40.62(1.46) | 41.10(1.86) | 41.22(1.42) |  |
| ≥60 | 16.62(1.30) | 23.07(1.37) | 27.03(1.59) | 31.08(1.50) |  |
| **sex** |  |  |  |  | **< .001** |
| Female | 43.96(1.05) | 48.08(1.50) | 55.48(1.29) | 58.19(1.44) |  |
| Male | 56.04(1.05) | 51.92(1.50) | 44.52(1.29) | 41.81(1.44) |  |
| **Race/ethnicity** |  |  |  |  | **< .001** |
| non-Hispanic White | 62.49(2.56) | 75.53(1.95) | 77.66(1.93) | 77.35(1.80) |  |
| non-Hispanic Black | 19.70(1.83) | 9.42(0.94) | 6.09(0.71) | 4.97(0.65) |  |
| Mexican American | 7.14(1.00) | 6.39(0.88) | 8.06(1.05) | 9.00(1.10) |  |
| others | 10.68(1.28) | 8.66(1.22) | 8.19(1.08) | 8.68(1.06) |  |
| **PIR** |  |  |  |  | 0.01 |
| low | 21.36(1.44) | 17.07(1.05) | 17.15(1.16) | 16.85(1.52) |  |
| middle | 39.26(1.38) | 37.49(1.64) | 34.71(1.54) | 37.00(2.02) |  |
| high | 39.38(1.99) | 45.44(1.74) | 48.14(2.20) | 46.15(2.48) |  |
| **BMI** |  |  |  |  | 0.17 |
| normal | 29.36(1.12) | 30.85(1.55) | 33.25(1.26) | 31.28(1.82) |  |
| overweight | 32.40(1.47) | 34.14(1.45) | 32.01(1.20) | 35.51(1.33) |  |
| obesity | 38.24(1.16) | 35.00(1.75) | 34.74(1.65) | 33.22(1.40) |  |
| **Marital status** |  |  |  |  | **< .001** |
| never married | 21.12(1.61) | 15.28(1.47) | 13.03(1.26) | 11.69(1.01) |  |
| married/living with partner | 62.27(1.76) | 66.35(1.70) | 68.44(1.68) | 68.93(1.61) |  |
| widowed/wivorced/separated | 16.61(1.02) | 18.37(1.10) | 18.53(1.18) | 19.38(1.10) |  |
| **Education** |  |  |  |  | **< .001** |
| college or more | 54.08(1.57) | 58.69(1.68) | 59.78(1.73) | 60.45(1.78) |  |
| middle school or lower | 5.42(0.75) | 4.21(0.48) | 4.82(0.57) | 7.33(0.77) |  |
| high school | 40.50(1.62) | 37.10(1.58) | 35.40(1.66) | 32.22(1.59) |  |
| **Home status** |  |  |  |  | **< .001** |
| owned or being bought | 67.95(2.15) | 73.14(1.61) | 76.35(1.61) | 77.38(1.82) |  |
| rented | 30.33(2.09) | 24.29(1.38) | 22.29(1.44) | 21.41(1.81) |  |
| **Smoke** |  |  |  |  | **< .001** |
| now | 27.88(1.26) | 23.10(1.39) | 19.85(0.98) | 14.91(0.82) |  |
| former | 21.20(1.41) | 26.70(1.44) | 25.87(1.07) | 30.07(1.37) |  |
| never | 50.92(1.74) | 50.20(1.87) | 54.28(1.46) | 55.02(1.54) |  |
| **Drinks** |  |  |  |  | 0.001 |
| nondrinkers | 29.17(1.53) | 26.45(1.34) | 30.64(1.63) | 30.20(1.66) |  |
| 1-3 drinks/day | 51.83(1.63) | 56.20(1.71) | 54.69(1.75) | 56.68(1.81) |  |
| ≥ 4 drinks/day | 19.00(1.10) | 17.35(1.13) | 14.67(0.99) | 13.12(1.00) |  |
| **Physical activity** |  |  |  |  | 0.3 |
| active | 39.65(1.34) | 41.10(1.74) | 38.70(1.71) | 38.89(1.87) |  |
| inactive | 26.85(1.35) | 25.72(1.34) | 25.32(1.50) | 27.20(1.62) |  |
| moderate | 12.00(0.92) | 14.77(0.88) | 13.52(1.12) | 14.24(1.34) |  |
| others | 21.51(1.18) | 18.41(1.25) | 22.47(1.53) | 19.67(1.16) |  |
| **CVD** | 7.45(0.85) | 7.77(1.00) | 8.56(0.94) | 9.69(0.81) | 0.29 |
| **DM** | 11.02(1.02) | 12.19(0.97) | 12.60(0.98) | 14.75(1.03) | 0.05 |
| **Hypertension** | 34.16(1.08) | 35.61(1.62) | 36.47(1.31) | 38.55(1.64) | 0.13 |
| **Cancer** | 7.83(1.22) | 9.99(1.05) | 9.59(0.97) | 10.22(0.74) | 0.34 |
| **Energy(kcal)** | 2097.27(37.71) | 2067.45(32.88) | 2045.52(26.69) | 1998.99(22.43) | 0.06 |
| **Healthy Dietary Score** | 48.05(0.45) | 50.42(0.42) | 52.66(0.48) | 54.94(0.49) | **< .001** |
| **Nitrate*** | 37571.66(1392.29) | 47064.99(1001.44) | 53066.15(980.99) | 71101.74(3158.01) | **< .001** |
| **Thiocyanate*** | 2435.62(152.12) | 2614.01(156.66) | 2602.10(108.72) | 2721.13(138.55) | 0.4 |
| **Year** |  |  |  |  | 0.01 |
| 2005-2006 | 35.30(2.48) | 37.36(2.56) | 33.57(2.10) | 29.66(2.11) |  |
| 2007-2008 | 28.75(2.51) | 28.70(2.12) | 32.14(2.30) | 38.60(2.80) |  |
| 2009-2010 | 35.95(2.58) | 33.95(2.18) | 34.28(1.90) | 31.74(2.79) |  |

Weighted Mean +/- Se and ANOVA for continuous variables. Weighted %, mean (95% CI), and Rao-Scott χ^2 test for categorical variables.

*Unit: μg/g creatinine.

**Supplementary Table 2.** Characteristics of the study participants by quartiles of nitrate.

|  | **Quartiles of** **Nitrate** | | | |  |
| --- | --- | --- | --- | --- | --- |
| **Variable** | **Q1** | **Q2** | **Q3** | **Q4** | **P value** |
| **Phenotypic age** | 45.01(0.61) | 41.02(0.53) | 41.98(0.71) | 44.41(0.70) | **< .001** |
| **Biological age** | 48.57(0.54) | 45.38(0.48) | 46.18(0.63) | 48.35(0.56) | **< .001** |
| **Age** | 47.82(0.55) | 45.34(0.50) | 46.68(0.62) | 49.52(0.58) | **< .001** |
| **Age groups** |  |  |  |  | **< .001** |
| <40 | 36.24(1.52) | 42.54(1.61) | 34.96(1.86) | 27.57(1.50) |  |
| 40-59 | 36.52(1.61) | 36.54(1.65) | 41.58(1.74) | 45.57(1.32) |  |
| ≥60 | 27.24(1.35) | 20.92(1.19) | 23.46(1.38) | 26.86(1.60) |  |
| sex |  |  |  |  | **< .001** |
| female | 43.46(1.12) | 44.10(1.42) | 52.91(1.27) | 64.00(1.29) |  |
| male | 56.54(1.12) | 55.90(1.42) | 47.09(1.27) | 36.00(1.29) |  |
| **Race/ethnicity** |  |  |  |  | **< .001** |
| non-Hispanic White | 65.85(2.35) | 71.38(2.09) | 75.84(1.89) | 79.62(1.84) |  |
| non-Hispanic Black | 19.95(1.76) | 11.05(1.23) | 5.81(0.72) | 4.24(0.52) |  |
| Mexican American | 7.11(0.92) | 8.29(1.13) | 8.55(1.08) | 6.56(0.88) |  |
| others | 7.10(0.92) | 9.28(1.10) | 9.80(1.10) | 9.58(1.14) |  |
| **PIR** |  |  |  |  | **< .001** |
| low | 18.06(1.21) | 18.89(1.36) | 19.00(1.32) | 16.25(1.14) |  |
| middle | 40.01(1.25) | 38.90(1.46) | 36.44(1.57) | 33.40(1.88) |  |
| high | 41.93(1.51) | 42.20(1.92) | 44.56(2.05) | 50.35(2.04) |  |
| **BMI** |  |  |  |  | **< .001** |
| normal | 23.83(1.22) | 27.44(1.31) | 33.03(1.68) | 39.31(1.58) |  |
| overweight | 33.56(1.85) | 35.30(1.46) | 31.93(1.11) | 33.32(1.03) |  |
| obesity | 42.60(1.67) | 37.27(1.48) | 35.04(1.60) | 27.36(1.18) |  |
| **Marital status** |  |  |  |  | **< 0.001** |
| never married | 18.92(1.25) | 16.97(1.58) | 13.99(1.32) | 11.49(1.08) |  |
| married/living with partner | 61.46(1.96) | 65.45(1.90) | 68.98(1.61) | 69.57(1.40) |  |
| widowed/divorced/separated | 19.61(1.57) | 17.59(1.35) | 17.03(1.10) | 18.94(0.98) |  |
| **Education** |  |  |  |  | 0.69 |
| college or more | 57.84(1.68) | 57.51(1.66) | 58.96(1.78) | 58.90(1.69) |  |
| middle school or lower | 5.82(0.64) | 4.85(0.56) | 5.25(0.60) | 5.76(0.60) |  |
| high school | 36.34(1.47) | 37.63(1.79) | 35.79(1.66) | 35.34(1.52) |  |
| **Home status** |  |  |  |  | **0.01** |
| owned or being bought | 72.26(1.77) | 72.57(1.46) | 72.27(1.81) | 77.80(1.37) |  |
| rented | 25.26(1.71) | 25.74(1.39) | 26.16(1.73) | 20.93(1.32) |  |
| **Smoke** |  |  |  |  | **< .001** |
| now | 11.11(1.03) | 19.55(1.36) | 27.72(1.47) | 25.43(1.18) |  |
| former | 27.62(1.19) | 26.60(1.51) | 23.45(1.04) | 26.68(1.40) |  |
| never | 61.27(1.53) | 53.85(1.74) | 48.84(1.53) | 47.88(1.57) |  |
| **Drinks** |  |  |  |  | **< 0.001** |
| nondrinkers | 32.44(1.74) | 28.04(1.39) | 26.88(1.62) | 29.49(1.39) |  |
| 1-3 drinks/day | 53.42(2.00) | 52.09(1.77) | 56.25(1.89) | 57.50(1.53) |  |
| ≥ 4 drinks/day | 14.14(1.10) | 19.86(1.53) | 16.87(1.01) | 13.01(0.96) |  |
| **Physical activity** |  |  |  |  | **< 0.001** |
| active | 34.15(1.64) | 44.38(1.48) | 38.44(1.77) | 40.74(1.93) |  |
| inactive | 27.63(1.28) | 24.61(1.34) | 26.55(1.49) | 26.32(1.94) |  |
| moderate | 13.18(1.06) | 11.99(0.87) | 14.55(0.99) | 14.80(0.98) |  |
| others | 25.04(1.36) | 19.02(1.43) | 20.46(1.08) | 18.14(1.33) |  |
| **CVD** | 10.45(0.78) | 7.15(0.90) | 7.68(0.69) | 8.46(0.91) | **0.03** |
| **DM** | 16.64(1.03) | 10.78(0.89) | 11.73(1.08) | 11.99(0.93) | **< .001** |
| **Hypertension** | 42.00(1.30) | 34.82(1.43) | 33.81(1.13) | 35.05(1.77) | **< 0.001** |
| **Cancer** | 11.42(0.97) | 8.20(0.97) | 8.50(0.95) | 9.88(0.93) | 0.07 |
| **Energy(kcal)** | 2018.42(28.25) | 2145.54(26.97) | 2055.27(28.77) | 1987.62(27.68) | **< .001** |
| **Healthy Dietary Score** | 49.65(0.48) | 49.57(0.43) | 51.74(0.46) | 54.88(0.46) | **< .001** |
| **Perchlorate*** | 3.17(0.10) | 4.37(0.15) | 4.78(0.11) | 7.06(0.22) | **< .001** |
| **Thiocyanate*** | 1209.31(61.91) | 2186.20(117.55) | 2865.45(126.74) | 3883.69(177.94) | **< .001** |
| **Year** |  |  |  |  | **0.02** |
| 2005-2006 | 37.56(2.74) | 32.50(1.95) | 33.95(1.74) | 32.53(2.77) |  |
| 2007-2008 | 26.51(2.23) | 32.40(2.15) | 31.99(2.05) | 36.33(2.57) |  |
| 2009-2010 | 35.93(2.52) | 35.10(2.07) | 34.06(1.93) | 31.13(2.34) |  |

Weighted Mean +/- Se and ANOVA for continuous variables. Weighted %, mean (95% CI), and Rao-Scott χ^2 test for categorical variables.

*Unit: μg/g creatinine.

**Supplementary Table 3.** Characteristics of the study participants by quartiles of thiocyanate.

|  | **Quartiles of** **Thiocyanate** | | | |  |
| --- | --- | --- | --- | --- | --- |
| **Variable** | **Q1** | **Q2** | **Q3** | **Q4** | **P value** |
| **Phenotypic age** | 46.00(0.78) | 42.78(0.66) | 42.47(0.53) | 41.56(0.53) | **< .001** |
| **Biological age** | 49.33(0.74) | 47.31(0.58) | 47.42(0.51) | 44.75(0.45) | **< .001** |
| **Age** | 49.20(0.74) | 47.63(0.56) | 47.62(0.49) | 45.32(0.43) | **< .001** |
| **Age groups** |  |  |  |  | **< .001** |
| <40 | 32.63(1.84) | 37.17(1.65) | 33.87(1.35) | 36.70(1.38) |  |
| 40-59 | 37.09(1.84) | 36.53(1.47) | 41.08(1.21) | 45.41(1.65) |  |
| ≥60 | 30.27(1.71) | 26.29(1.30) | 25.06(1.21) | 17.89(1.42) |  |
| sex |  |  |  |  | **0.02** |
| female | 47.91(1.76) | 53.79(1.35) | 53.99(1.39) | 49.76(1.31) |  |
| male | 52.09(1.76) | 46.21(1.35) | 46.01(1.39) | 50.24(1.31) |  |
| **Race/ethnicity** |  |  |  |  | **< .001** |
| non-Hispanic White | 63.06(2.55) | 71.01(2.05) | 77.74(1.70) | 79.87(1.73) |  |
| non-Hispanic Black | 12.47(1.31) | 9.28(0.91) | 8.80(0.99) | 9.37(1.11) |  |
| Mexican American | 11.73(1.51) | 9.31(0.95) | 6.64(0.89) | 3.81(0.59) |  |
| others | 12.74(1.52) | 10.39(1.23) | 6.83(0.78) | 6.94(0.93) |  |
| **PIR** |  |  |  |  | **< .001** |
| low | 19.23(1.67) | 15.42(0.93) | 13.33(1.00) | 24.28(1.75) |  |
| middle | 39.57(1.58) | 33.97(1.54) | 36.00(1.60) | 39.02(1.93) |  |
| high | 41.20(2.33) | 50.61(1.91) | 50.67(1.74) | 36.70(1.88) |  |
| **BMI** |  |  |  |  | **< .001** |
| normal | 28.77(1.35) | 28.85(1.36) | 28.89(1.45) | 37.80(1.25) |  |
| overweight | 35.57(1.46) | 33.10(1.23) | 34.35(1.24) | 31.42(1.35) |  |
| obesity | 35.66(1.73) | 38.06(1.45) | 36.76(1.42) | 30.77(1.18) |  |
| **Marital status** |  |  |  |  | **< 0.001** |
| never married | 17.61(1.04) | 15.37(1.43) | 13.16(1.07) | 15.08(1.34) |  |
| married/living with partner | 63.17(1.90) | 69.36(1.51) | 69.72(1.54) | 63.50(1.52) |  |
| widowed/divorced/separated | 19.23(1.45) | 15.27(0.90) | 17.12(1.06) | 21.41(1.14) |  |
| **Education** |  |  |  |  | **< .001** |
| college or more | 58.57(1.63) | 63.93(1.47) | 63.50(1.54) | 47.65(1.92) |  |
| middle school or lower | 8.97(0.96) | 5.73(0.66) | 3.51(0.42) | 4.20(0.57) |  |
| high school | 32.46(1.59) | 30.34(1.43) | 32.99(1.46) | 48.14(1.84) |  |
| **Home status** |  |  |  |  | **< .001** |
| owned or being bought | 73.02(2.03) | 76.33(1.18) | 77.42(1.85) | 68.42(1.43) |  |
| rented | 25.18(2.01) | 22.24(1.15) | 20.35(1.61) | 30.15(1.41) |  |
| **Smoke** |  |  |  |  | **< .001** |
| now | 2.18(0.44) | 4.77(0.62) | 11.69(0.88) | 61.96(1.59) |  |
| former | 29.36(1.54) | 28.23(1.69) | 30.46(1.40) | 16.86(1.12) |  |
| never | 68.45(1.51) | 67.00(1.56) | 57.85(1.73) | 21.19(1.20) |  |
| **Drinks** |  |  |  |  | **< .001** |
| nondrinkers | 36.99(1.66) | 29.46(1.65) | 27.49(1.59) | 24.11(1.47) |  |
| 1-3 drinks/day | 50.45(1.63) | 58.97(1.85) | 57.89(1.74) | 51.59(1.54) |  |
| ≥ 4 drinks/day | 12.56(0.97) | 11.57(0.89) | 14.62(1.44) | 24.31(1.09) |  |
| **Physical activity** |  |  |  |  | **0.003** |
| active | 35.24(1.60) | 37.19(1.66) | 41.77(1.75) | 43.14(1.73) |  |
| inactive | 27.14(1.42) | 27.41(1.59) | 25.84(1.39) | 24.81(1.51) |  |
| moderate | 12.60(0.90) | 15.05(1.05) | 14.27(0.97) | 12.60(1.20) |  |
| others | 25.03(1.42) | 20.35(1.34) | 18.12(1.30) | 19.45(1.32) |  |
| **CVD** | 11.37(0.89) | 7.45(0.85) | 7.51(0.92) | 7.72(0.78) | **0.004** |
| **DM** | 15.21(1.02) | 13.10(1.07) | 12.62(1.01) | 10.22(0.98) | **0.005** |
| **Hypertension** | 40.70(1.44) | 36.43(1.50) | 34.39(1.60) | 34.27(1.14) | **0.003** |
| **Cancer** | 8.93(0.77) | 10.82(1.14) | 9.48(0.73) | 8.50(0.76) | 0.16 |
| **Energy(kcal)** | 1983.90(21.81) | 2032.71(23.68) | 2060.42(32.47) | 2115.66(29.96) | **0.01** |
| **Healthy Dietary Score** | 51.96(0.43) | 53.27(0.46) | 52.66(0.50) | 48.51(0.48) | **< .001** |
| **Perchlorate*** | 4.22(0.15) | 4.76(0.17) | 5.43(0.20) | 5.11(0.17) | **< 0.001** |
| **Nitrate*** | 40822.68(1045.18) | 47525.45(1314.89) | 56170.65(2566.08) | 61964.57(1566.15) | **< .001** |
| **Year** |  |  |  |  | 0.12 |
| 2005-2006 | 36.37(2.49) | 33.35(2.18) | 32.87(1.94) | 33.92(2.45) |  |
| 2007-2008 | 29.15(2.03) | 30.13(2.11) | 33.50(2.03) | 34.62(2.51) |  |
| 2009-2010 | 34.48(2.21) | 36.52(2.04) | 33.63(1.97) | 31.46(2.64) |  |

Weighted Mean +/- Se and ANOVA for continuous variables. Weighted %, mean (95% CI), and Rao-Scott χ^2 test for categorical variables.

*Unit: μg/g creatinine.

**Supplementary Table 4.** Associations between PNT exposures and phenotypic age and biological age.

|  | **Phenotypic Age** | **Biological Age** |
| --- | --- | --- |
| character | β (95% CI) | β (95% CI) |
| **Perchlorate** | -0.42(-0.73, -0.10) | 0.14(-0.02, 0.30) |
| **Nitrate** | -0.43(-0.74, -0.12) | -0.3(-0.45, -0.14) |
| **Thiocyanate** | -0.51(-0.71, -0.30) | -0.14(-0.27, -0.01) |
| **Quartiles of Perchlorate** | | |
| Q1 | Ref. | Ref. |
| Q2 | -0.42(-0.93, 0.09) | 0.34(-0.01, 0.69) |
| Q3 | -0.55(-1.05, -0.05) | 0.33(-0.02, 0.68) |
| Q4 | -0.75(-1.29, -0.20) | 0.3(0.02, 0.57) |
|  | 0.01 | 0.081 |
| **Quartiles of Nitrate** | | |
| Q1 | Ref. | Ref. |
| Q2 | -0.48(-1.16, 0.21) | -0.39(-0.72, -0.07) |
| Q3 | -0.91(-1.48, -0.34) | -0.4(-0.68, -0.11) |
| Q4 | -0.64(-1.26, -0.02) | -0.61(-0.93, -0.29) |
|  | 0.016 | <0.001 |
| **Quartiles of Thiocyanate** | | |
| Q1 | Ref. | Ref. |
| Q2 | -0.74(-1.45, -0.02) | -0.02(-0.32, 0.28) |
| Q3 | -1.47(-2.03, -0.90) | -0.22(-0.54, 0.10) |
| Q4 | -1.19(-1.74, -0.64) | -0.23(-0.59, 0.13) |
|  | <.001 | 0.114 |

Models were adjusted for age, sex, ethnicity, PIR, BMI, marital status, home status, education, physical activity, smoke, drinks, hypertension, DM, CVD, cancer, energy(kcal), health dietary score, and NHANES cycle.

Ref.: reference.

**Supplementary Table 5.** Associations between PNT exposures and phenotypic age and biological age (Unweighted).

|  | **Phenotype Age** | **Biological Age** |
| --- | --- | --- |
| **character** | β (95% CI) | β (95% CI) |
| **Perchlorate** | -0.43(-0.68, -0.18) | 0.16(0.01, 0.32) |
| **Nitrate** | -0.97(-1.22, -0.73) | -0.77(-0.92, -0.62) |
| **Thiocyanate** | -0.69(-0.84, -0.53) | -0.29(-0.39, -0.19) |
| **Quartiles of Perchlorate** | | |
| **Q1** | ref | ref |
| **Q2** | -0.22(-0.64, 0.19) | 0.16(-0.10, 0.42) |
| **Q3** | -0.49(-0.91, -0.07) | 0.22(-0.04, 0.48) |
| **Q4** | -0.56(-0.99, -0.14) | 0.23(-0.04, 0.50) |
|  | 0.006 | 0.095 |
| **Quartiles of Nitrate** | | |
| **Q1** | ref | ref |
| **Q2** | -0.59(-1.00, -0.17) | -0.58(-0.84, -0.32) |
| **Q3** | -0.98(-1.39, -0.56) | -0.58(-0.85, -0.32) |
| **Q4** | -0.94(-1.36, -0.52) | -0.87(-1.13, -0.61) |
|  | <.001 | <.001 |
| **Quartiles of Thiocyanate** | | |
| **Q1** | ref | ref |
| **Q2** | -0.8(-1.22, -0.39) | 0(-0.26, 0.26) |
| **Q3** | -1.73(-2.15, -1.31) | -0.41(-0.68, -0.15) |
| **Q4** | -1.45(-1.96, -0.95) | -0.46(-0.78, -0.14) |
|  | <.001 | <0.001 |

Models were adjusted for age, sex, ethnicity, PIR, BMI, marital status, home status, education, physical activity, smoke, drinks, hypertension, DM, CVD, cancer, energy(kcal), health dietary score, and NHANES cycle.

Ref.: reference.
